# Endovascular Aneurysm Repair Versus Open Surgical Repair in Treating Abdominal Aortic Aneurysm

**DOI:** 10.7759/cureus.73066

**Published:** 2024-11-05

**Authors:** James R Vienneau, Camden I Burns, Anto Boghokian, Varun Soti

**Affiliations:** 1 Surgery, Lake Erie College of Osteopathic Medicine, Erie, USA; 2 Anesthesiology, Lake Erie College of Osteopathic Medicine, Elmira, USA; 3 Pharmacology and Therapeutics, Lake Erie College of Osteopathic Medicine, Elmira, USA

**Keywords:** abdominal aortic aneurysms (aaa), endovascular aortic repair (evar), open surgical repair (or), perioperative complications, postoperative mortality

## Abstract

Abdominal aortic aneurysm (AAA) denotes an abdominal aorta dilation exceeding 3 cm, typically asymptomatic until rupture, posing severe consequences, including fatality. Therefore, continual screening is imperative, and surgical intervention is recommended upon reaching a diameter of 5.5 cm to prevent rupture. The primary surgical approaches are open surgical repair or open repair (OR) and endovascular aneurysm repair (EVAR). This review juxtaposes EVAR’s short- and long-term effectiveness, safety, and perioperative complications in AAA patients versus OR, elucidating clinical benefits and avenues for further research. Following the Preferred Reporting Items for Systematic Reviews and Meta-Analyses guidelines, an extensive literature review was conducted using the PubMed and Clinicaltrials.gov databases. The review specifically focused on clinical studies directly comparing EVAR versus OR. The comprehensive literature review revealed that EVAR confers a survival advantage for up to four years post-procedure. However, the benefit shifts to OR after four to eight years due to aneurysm-related complications, such as ruptures, underscoring the necessity of lifelong post-EVAR monitoring. Following EVAR, AAA patients necessitate significantly more frequent secondary interventions due to graft-related issues, including endoleaks, thereby escalating long-term complexity and care costs. Conversely, following OR, a notably higher proportion of patients require mechanical ventilation and blood transfusions and experience prolonged intensive-care and mid-care unit stays, consequently extending hospitalization. After EVAR, patients recover substantially faster, returning to normal activities sooner. Nonetheless, the long-term quality of life between the two procedures becomes comparable. While EVAR presents itself as a less invasive alternative to OR, especially for high surgical risk patients, the imperative of long-term surveillance and the risk of secondary interventions pose significant challenges. Advancements in EVAR technology and technique are broadening its utility. Yet, continual research is crucial to optimize patient selection, improve long-term outcomes, and ensure that EVAR’s benefits outweigh the risks. Therefore, choosing EVAR over OR in treating AAA patients must factor in a patient’s overall health, anatomical considerations, and the probability of long-term success.

## Introduction and background

Abdominal aortic aneurysm (AAA) refers to a dilation of the abdominal aorta that exceeds 3 cm [1]. Most AAA patients are asymptomatic until the aneurysm ruptures. AAA rupture is typically fatal, with a mortality rate of 85% to 90%. Most patients who suffer AAA rupture do not make it to the hospital; however, for those who do make it, their survival rate is 50% to 70% [2]. AAAs are among the top 15 causes of death in the United States of America [3], accounting for roughly 13,000 deaths per year in the United States. These are more common in men than women, occurring in 8% of men and 2% of women aged over 65 [4]. The major risk factors contributing to AAAs are smoking history, biological sex, age, and family history of AAA [5]. Repair of an AAA is ranked in the top three for the highest percentage of perioperative mortality [6].

AAAs can develop anywhere between the diaphragm and the aortic bifurcation. There are three sub-classifications of AAA: suprarenal, pararenal, and infrarenal. Suprarenal aneurysms originate above the renal arteries, pararenal involves the renal arteries, and infrarenal originates below the renal arteries, accounting for approximately 80% of all AAAs [7]. The asymptomatic nature of AAA underscores the importance of screening in patient management. The United States Preventive Services Task Force (USPSTF) recommends that any male aged 65 to 75 years who has smoked more than 100 cigarettes in his lifetime should undergo a one-time abdominal ultrasound [8]. Additional diagnostic modalities include CT and MRI [9].

According to USPSTF data, the average size of an aneurysm upon diagnosis is 3.3 cm [10]. Per USPSTF recommendations, patients with AAA sizes between 3.0 and 3.9 cm should undergo screening every three years. Patients with aneurysm sizes between 4.0 and 4.4 cm must be screened every two years, while patients with aneurysm sizes between 4.5 and 5.4 cm should be screened annually. A smaller-sized AAA can be managed with more conservative approaches [11]. However, surgical repair is required when the aneurysm reaches a diameter of 5.5 cm, or it could rupture [12].

The pathophysiology of AAA is multifocal and not completely understood. There are several proposed mechanisms of injury, including neovascularization, chronic inflammation, proteolysis, and oxidative stress. Chronic inflammation leads to increasing numbers of macrophages and lymphocytes within the media and adventitia of the aorta, causing increased diameter of the aneurysm. It has previously been described that macrophages are among the most prevalent cells and secrete proteases that affect the aortic media. The cause of inflammation is not completely elucidated but has been proposed to be related to angiotensin II. Angiotensin II facilitates inflammatory effects by increasing leukocyte adhesion molecules and chemokine expression. Lymphocytes involvement in inflammation is associated with interleukins and tumor necrosis factor alpha release, underlying the local immune response. This combined inflammation ultimately leads to the apoptosis of smooth muscle cells and the loss of integrity of aortic media, increasing the risk of aneurysm development [13].

Another proposed mechanism of aortic aneurysm formation is proteolysis of extracellular matrix (ECM) proteins. Proteolysis creates an imbalance between the synthesis and destruction of ECM proteins, thus weakening and expanding aortic media and causing an aneurysm. Several proteases are involved in this process, with matrix metalloproteinase (MMP) being the predominant. It has been shown that AAA patients have elevated MMP-2 and MMP-9. Their increased levels have been demonstrated to be proportional to the diameter of an aortic aneurysm. MMPs are regulated by tissue inhibitors of metalloproteinases (TIMPs). It has been shown that AAA tissues have a smaller number of messenger ribonucleic acid of TIMPs. This mechanism illustrates that proteases degrade denatured collagen, decreasing the compliance of the aorta, thus allowing the formation and expansion of the aneurysm [14].

The major components that make up the aortic wall are elastin and collagen type I and III. Collagens are specifically responsible for the integral strength of the aortic wall. Within an aneurysm, there is an increased cellular turnover of collagen type III. Most of the newly synthesized collagen becomes concentrated within the aortic media. Increased collagen accumulation within the media disrupts the fibril formation in the aortic wall and reduces its tensile strength, which contributes to its expansion and ultimately results in aneurysm formation [14].

Furthermore, research has demonstrated that oxidative stress is critical in the development of AAA. Recent findings have emphasized a significant link between oxidative stress and the formation of AAA, which has important implications in comprehending aneurysm pathogenesis. Various factors can produce reactive oxygen species (ROS) and reactive nitrogen species (RNS), resulting in substantial damage to cells and tissues across various physiological states. Elevated levels of ROS and RNS have been observed in the aneurysm wall compared to the normal aorta and adjacent non-aneurysmal aortic wall [15].

Inflammatory cells infiltrating the aorta are the primary source of ROS production, including superoxide anion radicals and hydrogen peroxide, due to the increased activity of nicotinamide adenine dinucleotide phosphate hydrogen (NADPH) oxidase. Moreover, pro-inflammatory cytokines, mechanical stretch, growth factors, and lipid mediators may enhance NADPH oxidase in resident vascular cells, increasing ROS and lipid peroxidation products. Elevated ROS and RNS levels have been shown to upregulate the expression of MMPs through the activation of nuclear factor-kappa B and induce vascular smooth muscle cell apoptosis in the aneurysm wall [15].

AAA management is based on disease severity and multiple factors, including comorbidities. For example, if an initial screen reveals a small AAA, it can be effectively managed by using conservative treatment modalities such as incorporating routine exercise, following a healthy diet, and adhering to smoking cessation plans with continual follow-ups. On the other hand, if a patient has an AAA with comorbidities, for instance, hypertension, hyperlipidemia, and diabetes, a more comprehensive treatment approach encompassing appropriate pharmacological treatments is necessary [16-17].

Statins, the class of drugs that inhibit 3-hydroxy-3-methylglutaryl coenzyme A reductase, an enzyme involved in lipid metabolism, are typically used in AAA patients with hyperlipidemia. Research has shown that statins have additional benefits in slowing the progression of AAA. They achieve this by decreasing the secretion of MMPs and increasing the expression of TIMPs. Statins reduce the recruitment and accumulation of macrophages within the vascular wall; it occurs through the blockage of intercellular adhesion molecule-1 (ICAM-1) and monocyte chemoattractant protein-1 (MCP-1). This disruption of the pathogenesis underlying AAA formation by statins provides therapeutic benefits to AAA patients [18].

Angiotensin-converting enzyme (ACE) inhibitors and angiotensin II receptor blockers (ARBs) have demonstrated favorable outcomes in patients with AAA. ACE inhibitors mitigate the risk of aneurysm rupture by impeding aneurysm expansion through the inhibition of ICAM-1 and MCP-1. ARBs have exhibited the ability to reduce the activity of nuclear factor-kappa B and the expression of MMPs, thereby conferring significant advantages in averting AAA expansion and consequentially lowering the risk of rupture [19-22].

Another category of medications, known as non-steroidal anti-inflammatory drugs (NSAIDs), such as celecoxib, has gained significant attention in recent years. Celecoxib functions by inhibiting cyclooxygenase-2, which plays a role in promoting prostaglandin production in macrophages. This, in turn, upregulates MMPs, contributing to the formation and progression of AAAs. However, the clinical efficacy of these drugs has yet to be established, as there are currently no clinical trials evaluating the effectiveness of these drugs, particularly celecoxib, in the treatment of AAA, therefore augmenting the need for more research [23-24].

When pharmacological treatments prove ineffective or when the diameter of an AAA reaches a critical point exceeding 5.5 cm, surgical intervention becomes necessary. Two commonly employed surgical procedures for this purpose are open surgical repair or open repair (OR) and endovascular aneurysm repair (EVAR). OR involves making an abdominal incision, clamping the vessels above and below the aneurysm, opening the aneurysm sack, and inserting a synthetic graft. The graft is then secured to the normal aorta above and below [25].

On the other hand, EVAR is a meticulous procedure that begins with gaining access to the bilateral common femoral arteries, achieved through incision or percutaneously. Sheaths are then placed in the arteries, and through repeated imaging, the endovascular device is advanced to the AAA location as determined by preoperative CT angiography. Once in place, a stent is deployed, and its placement is confirmed via imaging, ensuring the absence of endoleaks around it [26].

Given the increasing preference for EVAR over OR in the surgical treatment of AAA, this review article provides a comprehensive analysis of the current literature to compare the efficacy and safety of EVAR versus OR. It also evaluates the incidence of intra- and post-procedural complications in AAA patients undergoing EVAR versus OR. Furthermore, this systematic review aims to identify research gaps to encourage further investigation and highlight the clinical benefits of each procedure, ultimately assisting cardiovascular surgeons and healthcare professionals in making well-informed surgical decisions and optimizing care for AAA patients.

## Review

Methods

**Figure 1:**
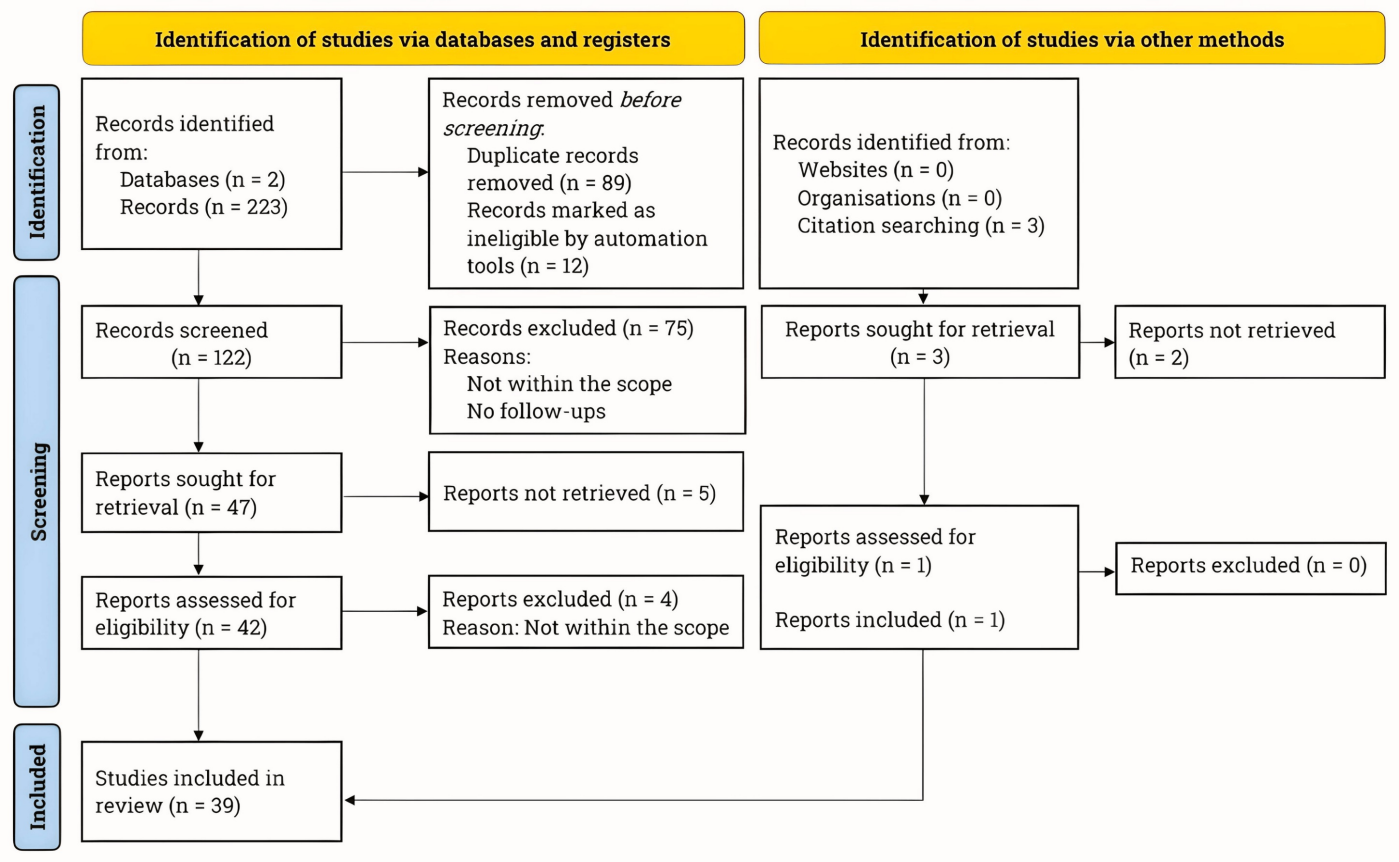
PRISMA flowchart for literature search. We adhered to the PRISMA guidelines and conducted an extensive literature review using PubMed and Clinicaltrials.gov databases. Our primary emphasis was on studies examining the short- and long-term safety, efficacy, perioperative complications, and quality of life of patients with abdominal aortic aneurysms after undergoing endovascular aneurysm repair and open surgical repair. n, number; PRISMA, Preferred Reporting Items for Systematic Reviews and Meta-Analyses

The literature search was conducted between January and August 2024 using PubMed and Clinicaltrials.gov databases in accordance with the Preferred Reporting Items for Systematic Reviews and Meta-Analyses (PRISMA) guidelines [27]. Figure 1 presents the PRISMA flowchart illustrating the literature search process.

The key search terms included “Abdominal Aortic Aneurysm”, “Abdominal Aortic Aneurysm Classification”, “Aortic Aneurysm, Abdominal Diagnosis”, “Abdominal Aortic Aneurysm Diagnostic Imaging”, “Abdominal Aortic Aneurysm Pathology”, “Abdominal Aortic Aneurysm Surgery”, “Vascular Surgical Procedures”, “Endovascular Aneurysm Repair”, and “Endovascular Procedures.” For more details, please see the Appendix.

The primary focus of the literature search was identifying and selecting studies addressing the efficacy, perioperative outcomes, postoperative complications, quality of life (QoL), short-term and long-term outcomes, and mortality rates associated with EVAR and OR for AAAs. The inclusion criteria for the studies are detailed in Table 1, with a specific emphasis on comparative assessments of the safety and effectiveness of EVAR versus OR and the stipulation that selected studies be published in English.

**Table 1:** Study selection criteria. The table presents the inclusion criteria used for the study selection in this systematic review. We meticulously considered studies that directly compared the safety and efficacy of EVAR with OR in treating AAA patients. Also, we focused on studies that assessed the screening, diagnosis, and pharmacological and surgical treatment approaches for AAA management. Our rigorous process ensured that only the most relevant and reliable studies published in English, meeting the criteria outlined in the table, were given higher priority. AAA, abdominal aortic aneurysm; EVAR, endovascular aneurysm repair; OR, open repair

Inclusion criteria for studies directly comparing the safety and efficacy of EVAR versus OR in treating AAA patients	Inclusion criteria for studies focused on AAA subclassification, pathobiology, screening, diagnosis, pharmacological treatment, and surgical approaches
Randomized controlled trials	Meta-analyses
Non-randomized controlled trials	Systematic reviews
Prospective clinical studies	Narrative reviews
Observational studies	Commentaries
Comparative studies	Opinions
Pilot studies	Pre-clinical studies
Case series	All study types mentioned in the left column
Case reports	

A total of 39 studies were included in this review. Table 2 provides a breakdown of the number of studies included in this review, grouped by category.

**Table 2:** The number of studies reviewed. The table presents the breakdown of the number of studies in this review grouped by category. AAA, abdominal aortic aneurysm; EVAR, endovascular aneurysm repair; OR, open repair

Category	Number of studies included
AAA screening, diagnosis, and pharmacological treatment	19
AAA subclassification and pathophysiology	4
AAA surgical procedures	3
Method for systematic literature search	1
Clinical evidence of direct comparison of EVAR versus OR in AAA patients	12
	Total studies = 39

Clinical evidence of the safety and efficacy of EVAR versus OR in treating AAA patients

Prinssen et al. conducted a multicenter, double-blind, randomized controlled trial to compare the 30-day mortality rates and perioperative outcomes associated with OR and EVAR in patients diagnosed with AAA. The primary objective was to assess the efficacy of these two surgical approaches, with secondary objectives focused on discerning differences in perioperative outcomes between the procedures. A total of 351 patients participated, with 173 patients assigned to undergo EVAR and 178 patients to undergo OR. However, two patients from the EVAR group and four from the OR group did not undergo the respective procedures, resulting in 345 patients who completed the surgeries. Participants were required to have an AAA with a minimum diameter of 5 cm and to be eligible for either EVAR or OR [28].

The study revealed that EVAR was associated with significantly shorter operation times, reduced intraoperative blood loss, fewer blood transfusions, and lower rates of mechanical ventilation than OR (p<0.001). Although the risk of operative mortality was higher in the OR group (n=8) than in the EVAR group (n=2), with a risk ratio (RR) of 3.9, it did not reach statistical significance (p=0.10). Systemic complications, particularly pulmonary complications, were also more prevalent in the OR group (p<0.001 and p=0.005, respectively). Conversely, local vascular or implant-related complications were more frequent in the EVAR group than in the OR group (p=0.03) [28].

Regarding secondary outcomes, the study reported that the mean duration of the EVAR procedure was 135 minutes, significantly shorter than the 151 minutes required for OR (p<0.001). Additionally, patients in the EVAR group experienced a markedly lower mean blood loss (394 mL). They required fewer blood transfusions (0.09 units) than those in the OR group, who experienced an average blood loss of 1,653 mL and required 0.44 units of blood (both p<0.001). Furthermore, the duration of stay in the medium care unit (MCU) or ICU was significantly shorter for the EVAR group, averaging 16 hours, compared to 72 hours for the OR group (p<0.001) [28].

The study concluded that EVAR is associated with a lower incidence of mortality, shorter procedure times, and reduced ICU/MCU stays compared to OR, suggesting that EVAR may be the preferred surgical option for AAA treatment. The results underscore the efficacy of EVAR in achieving superior perioperative outcomes, including reduced blood loss and transfusion requirements, thereby advocating its adoption as a standard treatment for AAA [28].

Greenhalgh et al. investigated the 30-day mortality rates associated with EVAR and OR in AAA patients in a randomized controlled trial. The study also evaluated postoperative complications, aneurysm-related mortality, secondary interventions, hospital stay duration, and operative time. The cohort initially consisted of 2,068 patients with AAAs that were 5.5 cm or larger in diameter and had suitable anatomy for both procedures. However, the researchers could enroll 1,082 eligible patients and randomly assigned them to two groups: 543 patients underwent EVAR, while 539 underwent OR. Accounting for preoperative losses, 512 patients underwent successful EVAR, and 496 completed OR. The patient population included individuals with comorbidities such as diabetes, smoking history, cardiac disease, and hypertension, with no statistical differences noted between the groups. The study reinforced the favorable outcomes of EVAR in managing AAA [29].

The study revealed that 30-day mortality was significantly higher in the OR group (n=24) than in the EVAR group (n=9) (p=0.016). In-hospital mortality rates also favored EVAR, with 32 deaths in the OR group versus 11 in the EVAR group (p=0.001). However, secondary interventions within 30 days were more frequent in the EVAR group (n=52) than in the OR group (n=30) (p=0.02). Additionally, patients who underwent OR required longer hospitalization and had extended operative times (p<0.0001) [29].

Concerning the secondary results, the EVAR procedure averaged 180 minutes, significantly shorter than the 200 minutes required for OR (p<0.0001). Hospital stays were also shorter for EVAR patients, with an average of seven days compared to 12 days for OR patients (p<0.0001). Further analysis showed that EVAR patients were more likely to require conversions to open repair (n=10), correction of endoleaks (n=18), or other procedures (n=23) compared to the OR group (n=0, n=1, n=14, respectively). Conversely, re-exploration of the surgical site was more common in OR patients (n=15) than in those who underwent EVAR (n=1) [29].

These findings suggest that EVAR is associated with lower mortality rates, shorter hospital stays, and shorter procedure times than OR in the treatment of AAA. However, the need for secondary interventions was higher in the EVAR group, highlighting the trade-off between short-term advantages and potential long-term complications. The study provides further evidence supporting the preferential use of EVAR for AAA, albeit with a noted increase in secondary interventions [29].

Greenhalgh et al., leading the EVAR trial participants, extended their randomized controlled trial to a four-year follow-up period to evaluate long-term survival rates between EVAR and OR. The study included the same cohort of 1,082 patients from the Greenhalgh et al.'s study, with secondary objectives focusing on secondary interventions, blood product usage, contrast agent use, length of hospital stay, and health-related quality of life (HRQL). HRQL assessment incorporated the physical and mental components of the 36-Item Short Form Health Survey (SF-36) and EuroQol 5-D (EQ5D) at one, three, and 12 months post-procedure. This study provided valuable insights into the long-term outcomes of EVAR and OR in managing AAA [30].

After four years of follow-up, the study showed no significant difference in overall mortality between the OR (n=109) and EVAR (n=100) groups (p=0.46). However, aneurysm-related mortality was significantly lower in the EVAR group (n=19) than in the OR group (n=34) (p=0.04). Similar to earlier findings, OR was associated with higher blood product usage and extended hospital stays (p<0.05). The rate of secondary procedures was higher in the EVAR group (41%) than in the OR group (9%) (p<0.0001). The study also reported that patients who underwent EVAR experienced a higher QoL in the first three months post-procedure, as measured by EQ5D and SF-36 physical components, than those who underwent OR (p=0.01 and p=0.001, respectively), though the patients could not sustain this difference in later follow-ups (EQ5D: p=0.37, p=0.29; SF-36: p=0.91, p=0.71). The study also highlighted the higher long-term costs associated with EVAR, primarily due to the need for more frequent follow-ups and repeat CT scans to ensure graft durability [30].

Secondary outcomes of the study indicated that the EVAR procedure was quicker, with an average duration of 182 minutes, compared to 205 minutes for OR (p<0.05). EVAR patients also required significantly fewer blood products (164 mL) than OR patients (896 mL) (p<0.01). Additionally, OR patients spent more time in the ICU/cardiac ICU (2.4 days), high-dependency unit (1.9 days), and both preoperative and postoperative wards (9.2 days and 2.2 days, respectively) compared to EVAR patients (0.7, 0.9, 6.9, and 1.9 days, respectively). The overall hospital stay was also longer for OR patients (15.7 days) than for EVAR patients (10.3 days) (p<0.05) [4].

The findings of this study further support the short-term survival advantage and efficacy of EVAR in AAA treatment, which is consistent with previous research. However, this study also emphasizes the need for extended follow-up to fully assess the long-term efficacy of EVAR, as the initial benefits appear to diminish over time. A notable limitation of the study was the lack of statistical analysis for some secondary outcomes [30].

In a subsequent randomized controlled trial, Patel et al. (2016) conducted a comparative analysis of total and aneurysm-related mortality rates associated with EVAR and OR. The focus was on both short- and long-term outcomes. The study categorized the results across different time frames, including six months, six months to four years, four years to eight years, and beyond eight years. A total of 1,252 patients were enrolled and equally assigned to the two treatment groups. This study extended the work of Greenhalgh et al., which included 1,082 patients from the original cohort and incorporated 175 newly recruited patients who met the same eligibility criteria. Participants were required to have an AAA with a minimum diameter of 5.5 cm, be at least 60 years old, and be suitable candidates for EVAR and OR [31].

The study revealed a slightly higher, but statistically non-significant, overall mortality following EVAR (n=466, 74%) than following OR (n=450, 72%) (p=0.41). However, aneurysm-related mortality was significantly higher in the EVAR group (n=84, 13%) than in the OR group (n=52, 8%) (p=0.01), particularly beyond the 4-year mark. During the first six months post-procedure, there was no significant difference in aneurysm-related mortality between EVAR (n=3, 0.5%) and OR (n=4, 0.7%) (p=0.70). However, between four and eight years, aneurysm-related mortality was higher in the EVAR group (p=0.001) and continued to increase beyond eight years (p=0.02). The study also reported higher rates of aneurysm rupture in the EVAR group than in the OR group (p=0.0001) and a greater need for secondary interventions (p=0.002) [31].

Secondary outcomes revealed a mean procedure duration of 186 minutes for EVAR, significantly shorter than the 202 minutes for OR (p=0.0002). ICU and hospital stays were also shorter for EVAR patients (1.2 and 10.8 days, respectively) than for OR patients (3.2 and 15.5 days, respectively) (p=0.0001 for both). The study noted a significant advantage for EVAR regarding early postoperative outcomes, including reduced blood loss and faster recovery. Still, it underlined the higher long-term risk of aneurysm-related complications and mortality associated with EVAR [31].

In a comparative study, Salata et al. contrasted the long-term outcomes of EVAR with OR in AAA patients. Based on a population-based cohort, the study included 17,683 patients aged 40 and above who underwent elective EVAR or OR for AAA repair. The primary outcome was overall survival, while secondary outcomes involved recording significant adverse cardiovascular event-free survival, reintervention, and secondary rupture [32].

About 34.5% underwent EVAR and 65.5% underwent OR. The average follow-up was four and four-tenths years, with a maximum follow-up of 13.8 years. The study findings showed that compared with OR, EVAR was associated with higher survival rates up to one year after repair (91.0% versus 94.0%) and higher significant adverse cardiovascular event-free survival rates up to four years after repair (69.9% versus 72.9%). The cumulative incidence of reintervention was higher among EVAR patients than among OR patients at the seven-year follow-up (45.9% versus 42.2%) [32].

However, the study revealed no statistically significant differences between EVAR and OR patients in long-term survival, reintervention, or secondary rupture. The Kaplan-Meier estimator indicated better long-term survival free of major adverse cardiovascular events in EVAR patients than those with OR, with a maximum follow-up of 13.8 years. The study concluded that EVAR was not associated with differences in long-term survival during more than 13 years of maximum follow-up [32].

In a multicenter randomized controlled trial, De Bruin et al. determined the long-term mortality rates among patients undergoing EVAR and OR for AAA. The study assessed each procedure’s efficacy and associated complications, with secondary objectives focusing on reintervention rates, categorized into graft-related indications, interventions related to the incision site, and local or systemic vascular complications. A total of 351 patients were enrolled, with 178 assigned to OR and 173 to EVAR [33].

Eligibility criteria required patients to have an AAA of at least 5 cm in diameter and be suitable candidates for both procedures. Exclusion criteria included the need for emergency surgery, the presence of an inflammatory aneurysm, anatomical variations, connective tissue disorders, or previous organ transplants. The study monitored patients at 30 days, six months, 12 months, 18 months, 24 months, and six years after their initial procedure. The study’s findings provided insight into EVAR's long-term efficacy and safety versus OR in managing AAA [33].

The study revealed no significant difference in overall survival between the procedures over six years of follow-up despite a slightly higher number of deaths in the OR group (n=60) than in the EVAR group (n=58) (p=0.97). However, in-hospital mortality was notably higher following OR (n=8) than EVAR (n=2) (p<0.0001). Cardiovascular-related deaths were more frequent among OR patients (n=20) than EVAR patients (n=16) (p<0.05). Additionally, reintervention was more common in the EVAR group (n=48) than in the OR group (n=30) (p=0.03) [33].

Further analysis of secondary outcomes indicated that graft-related issues were the most frequent cause for reintervention following EVAR (n=36, 75%). In contrast, wound-related complications, particularly incisional hernia (n=14, 47%), were more common after OR. Local vascular or systemic complications were more prevalent in the OR group (n=11) than in the EVAR group (n=9). These findings reinforce the efficacy and safety of EVAR, suggesting it is a more effective surgical option than OR for AAA treatment. The lack of significant differences in six-year mortality challenges the findings of Patel et al., who reported a slight long-term survival benefit with OR. The short-term survival advantage of EVAR, combined with these results, strengthens the case for EVAR as the preferred approach for AAA management [33].

Lederle et al. extended the follow-up period to 14 years in another randomized controlled trial to evaluate the long-term efficacy, safety, and differential outcomes between EVAR and OR. This study involved 881 patients, 444 undergoing EVAR and 437 undergoing OR. Inclusion criteria mirrored those of previous studies, requiring an AAA of at least 5 cm in diameter with patients suitable for EVAR. Their findings further support EVAR as a preferred surgical option for AAA [34].

The results showed that overall mortality was slightly lower in the EVAR group (n=304) than in the OR group (n=306), with a hazard ratio of 0.96 (p=0.61). Mortality within the first six months and between six months and four years was lower in the EVAR group (n=11, n=59, respectively) than in the OR group (n=14, n=70, respectively) (p=0.51, p=0.22, respectively). However, a slight survival advantage was observed in the OR group (n=76) during the four- and eight-year follow-up period compared to the EVAR group (n=93) (p=0.29). OR patients (n=36) exhibited a 2.8% higher incidence of death due to pulmonary complications than EVAR patients (n=24). Further analysis indicated a 2.1% higher risk of aneurysm-related death in OR patients (n=11) within the hospital or 30 days postoperatively compared to EVAR patients (n=2). Cardiovascular-related deaths were 4% higher in the EVAR group (n=88) than in the OR group (n=69). Additionally, secondary interventions were more common in the EVAR group (n=117) than in the OR group (n=85) (p=0.04). The secondary analysis highlighted that patients over 70 years of age had better overall survival following OR (p=0.02), whereas those under 70 years had higher survival rates with EVAR (p=0.10) [34].

These findings suggest EVAR offers a survival advantage in the early postoperative years. However, the study emphasizes that OR is associated with more aneurysm-related deaths [34], aligning with the results of De Bruin et al., which also indicated a higher need for secondary interventions following EVAR [33]. This study underscores the survival differences based on age, with a critique being the lack of statistical analysis on specific causes of death. The findings advocate for EVAR’s minimally invasive approach, particularly in younger patients, as a potential benefit in AAA treatment [34].

van Schaik et al. conducted a prospective randomized controlled trial to compare long-term mortality and postoperative outcomes between OR and EVAR, focusing on all-cause mortality and the necessity of secondary interventions. The study included 351 patients, 173 undergoing EVAR and 178 undergoing OR, although four OR patients and two EVAR patients did not complete the procedure. Preoperative age, comorbidities, and medications were similar across the groups, except for a higher prevalence of pulmonary disease in the EVAR group (27.7%) than in the OR group (18.5%) (p=0.04). The researchers followed up on patients for up to 15 years after the initial procedure. The study’s findings contribute valuable data on the comparative efficacy of the two procedures, aiding in clinical decision-making for AAA management [35].

Results indicated that EVAR patients (n=6) experienced lower mortality within the first six months postoperatively than OR patients (n=10), with a relative risk (RR) of 1.65 (p=0.34). Between six months and six years postoperatively, OR patients (n=43) showed a slightly lower mortality risk than EVAR patients (n=48), with an RR of 0.83. There were no significant differences in mortality between the procedures beyond six years (p=1.0). However, secondary interventions were more frequently required in the EVAR group (n=99) than in the OR group (n=44), with an RR of 0.42 (p<0.001). Aneurysm-related complications were more common in the EVAR group, including incomplete aneurysm exclusion (p<0.001) and thrombo-occlusive events (p<0.01). Conversely, incisional hernias were more prevalent following OR (p=0.006) [35].

Secondary outcomes revealed that pulmonary complications leading to death were significantly more frequent in the OR group, with an RR of 6.01 (p<0.01). Aneurysm-related deaths within the first six months postoperatively were also more common in OR patients, with an RR of 3.31 (p=0.06). This increased risk persisted throughout follow-up, with an RR of 1.54 (p=0.35) [35].

The findings from this study align with previous research, suggesting that EVAR offers superior short-term outcomes. The increased frequency of secondary procedures following EVAR may contribute to the observed reduction in aneurysm-related deaths. However, the study’s large follow-up intervals are a limitation, with more frequent interval analysis potentially providing more reliable statistical insights. Overall, these results further underscore the potential benefits of EVAR, solidifying its position as the optimal surgical option for AAA treatment [35].

In their multicenter randomized controlled trial with a three-year follow-up, Becquemin et al. compared the all-cause mortality and secondary intervention rates between EVAR and OR. The study evaluated the efficacy of EVAR and OR, identified associated complications, elucidated differences in patient outcomes, found that EVAR was less invasive, and demonstrated efficacy and safety in managing AAA [1]. The researchers randomized 299 patients into two groups: 149 underwent OR, while 150 underwent EVAR. Seventeen patients in the OR group and four in the EVAR group crossed over to the other procedure, predominantly due to patient preference, with a significant number switching from OR to EVAR (p<0.01). Eligibility criteria included an AAA with a diameter of at least 5 cm in men or 4.5 cm in women [36].

The results indicated that patients who underwent OR had significantly more extended hospital stays, required more RBC transfusions, and needed ventilatory support more frequently (p<0.0001). There was no significant difference in operative mortality between the groups (p=0.24). However, 16% of EVAR patients required vascular reintervention, compared to 2.7% of those who underwent OR (p<0.0001). Conversely, incisional and minor cardiac complications were more common in the OR group, with p-values of <0.0001 and 0.05, respectively. Additionally, postoperative buttock claudication was more frequent in the EVAR group (p<0.001). Although the risk of postoperative sexual dysfunction was higher in the OR group, this difference was not statistically significant. The findings underscore the safety of EVAR in managing AAA [36].

The secondary outcomes further demonstrated that the OR procedure took an average of 2.8 hours, significantly longer than EVAR, which averaged 2.1 hours (p<0.0001). Patients who underwent OR required substantially more blood transfusions, averaging 2.1 units of packed RBCs, compared to 0.2 units in the EVAR group (p<0.0001). Additionally, OR patients had more extended hospital stays, averaging 10.4 days, and required more extended postoperative ventilatory support, averaging 8 hours, compared to 5.8 days and 3.2 hours, respectively, in the EVAR group (p<0.0001 for both). The efficiency of EVAR is evident in these results, instilling confidence in its use for AAA patients [36].

Hwang et al. conducted another randomized controlled trial to compare mortality rates and postoperative outcomes between OR and EVAR. The study enrolled 240 patients with a non-ruptured AAA of at least 5 cm diameter. It randomized them into three groups: 146 underwent OR, 42 underwent EVAR under anatomically compliant conditions per manufacturer's instruction for use (IFU EVAR), and 52 underwent EVAR under non-compliant conditions (non-IFU EVAR). The primary preoperative difference was that OR patients were significantly younger than those in the EVAR groups (p=0.003). The study provided further insights into managing AAA patients, highlighting the procedural efficacy [37].

The findings revealed that reinterventions were more frequent in patients undergoing IFU EVAR and non-IFU EVAR than in those undergoing OR (p=0.02). However, there was no significant difference in reinterventions between IFU EVAR and OR (p=0.881). Patients in the non-IFU EVAR group had significantly more type I endoleaks than those in the IFU EVAR group (p=0.004). Additionally, non-IFU EVAR patients exhibited higher rates of type IA and IB endoleaks than IFU EVAR patients, with p-values of 0.162 and 0.058, respectively. The mortality rates at three- and five-year follow-ups were highest among non-IFU EVAR patients (44% and 55%), while OR patients had 22% and 29% mortality rates, respectively. IFU EVAR patients had the lowest mortality rates (14% and 23%) (p=0.098) [37].

These findings underscore the benefits of EVAR, particularly when performed according to manufacturer specifications. The results demonstrated lower reintervention rates and overall mortality in IFU-EVAR patients than in OR patients, emphasizing the importance of adhering to manufacturer guidelines. This study adds to the body of evidence supporting EVAR as the preferred surgical approach for AAA patients, especially when anatomical criteria are met [37].

Cuypers et al. investigated the mortality and cardiac complications associated with OR and EVAR in a related study. The study focused on all-cause mortality, cardiac death, congestive heart failure, myocardial infarction, and other cardiac events, assessed through ST abnormalities on ECG and cardiac wall motion abnormalities on transesophageal echocardiography (TEE). The eligibility criteria included patients with an AAA of at least 5 cm in diameter who were candidates for both EVAR and OR. The researchers enrolled 76 patients, 57 undergoing EVAR and 19 undergoing OR, and followed them up for up to 30 days after procedures. This study provided further insights into the hemodynamic and cardiac responses following each procedure, adding to the evidence supporting EVAR as the preferred option for AAA patients [38].

The results indicated that EVAR patients had significantly shorter hospital stays and a quicker return to regular feeding than OR patients (p<0.01). OR patients required longer intubation and spent more time in the ICU (p<0.05). They also had a higher need for transfusions (p<0.05). The incidence of postoperative ST-segment abnormalities on ECG was 42% in OR patients, compared to 18% in EVAR patients (p=0.06). OR patients also had a higher risk of myocardial infarction (p=0.05). Additionally, heart wall motion abnormalities on TEE were more frequent in OR patients (28%) than in EVAR patients (13%). Perioperative left ventricular stroke work index (SWI) and blood pressure (BP) were higher in OR patients, with respective p-values of 0.01 and 0.02 [38].

Secondary outcomes revealed that OR patients had an average hospital stay of 11 days, while EVAR patients stayed for five days (p<0.01). OR patients also spent more time in the ICU (21 hours) and required longer intubation (10 hours) than EVAR patients (19 hours in ICU, seven hours intubated). Additionally, OR patients took longer to resume regular feeding, averaging five days compared to two days in EVAR patients (p<0.01). Packed RBC transfusions were twice as frequent in OR patients (two units) compared to EVAR patients (zero units) [38].

These findings further substantiate the minimal invasiveness of EVAR, highlighting its safety and effectiveness. EVAR was associated with shorter hospital stays, reduced intubation time, fewer RBC transfusions, and a quicker return to a normal diet than OR. Additionally, EVAR demonstrated lower perioperative SWI and BP, indicating less operative stress. The study also showed fewer postoperative ECG or TEE abnormalities and fewer myocardial infarctions in EVAR patients. However, the small sample size limits the generalizability of these results, although they align with existing evidence supporting EVAR as the preferred surgical option for AAA treatment [38].

Soulez et al. conducted a randomized controlled trial to assess functional autonomy, QoL, and pain control after EVAR and OR. Forty patients were randomized into two groups: 20 underwent EVAR, and 20 underwent OR. Eligibility criteria included patients under 80 years of age with an AAA of at least 5 cm in diameter located below the renal arteries. The study evaluated patients one, six, and 12 months after the initial procedure. Functional autonomy was assessed using Karnofsky scores, and QoL was measured using SF-36. This study provided a comparative analysis of the efficacy of EVAR and OR, offering insights into the clinical management of AAA patients [39].

The study demonstrated that EVAR patients had significantly shorter hospital and ICU stays, required fewer transfusions, and experienced shorter procedure times. Additionally, patients undergoing EVAR had fewer rehospitalizations for any cause and aneurysm-related issues (p=0.18 and 0.55, respectively) than those undergoing OR. There were no significant differences in QoL or functional autonomy between the groups, as measured by Karnofsky and SF-36 scores. However, OR patients had significantly higher morphine consumption postoperatively (p<0.05) [39].

Analysis of secondary outcomes revealed that EVAR patients spent less time in the ICU (3.4 hours) and had shorter hospital stays (4.5 days) than OR patients (38.5 hours in the ICU and 11.5 days in the hospital; p=0.0001 and p=0.001, respectively). OR patients also required more RBC transfusions (1.1 units) than EVAR patients, who needed none (p=0.02). Additionally, the median procedure time was significantly longer for OR patients (230 minutes) than for EVAR patients (114 minutes; p=0.01). Despite these differences, the groups' overall QoL and functional autonomy scores were similar. However, OR patients had a higher demand for morphine, indicating more severe postoperative pain (p<0.05) [39].

These findings further corroborate the reduced invasiveness of EVAR and its associated benefits, including shorter ICU and hospital stays, fewer RBC transfusions, and shorter procedure times. The study did not find significant differences in QoL or functional autonomy between the groups, indicating that both procedures effectively restored patients to their preoperative status. However, the increased morphine consumption in OR patients suggests more significant postoperative pain, which may affect recovery. These results provide further evidence supporting the use of EVAR in suitable patients, especially considering its reduced perioperative burden [39]. Table 3 highlights a summary of the primary attributes of the studies examined in this subsection and the condensed findings.

**Table 3:** Summary of key studies directly comparing the safety and efficacy of EVAR versus OR in treating patients with abdominal aortic aneurysms. The table showcases the key characteristics of the studies exploring the safety and efficacy of EVAR versus OR in treating patients with abdominal aortic aneurysms and highlights critical findings. BP, blood pressure; EVAR, endovascular aneurysm repair; IFU, instruction for use (anatomical compliance per manufacturer specifications); OR, open repair; QoL, quality of life; RCT, randomized controlled trial; RR, relative risk; SF-36, 36-Item Short Form Health Survey; SWI, stroke work index; TEE, transesophageal echocardiography

Authors	Type of study	Sample size	Length of follow-up	Findings
Prinssen et al. (2004) [28]	RCT	351	30 days	The study findings revealed that patients who underwent EVAR had notably reduced operative times, minimized bleeding, fewer blood transfusions, and lower rates of mechanical ventilation than those who underwent OR (p<0.001). However, EVAR was associated with a higher frequency of complications related to local vascular implants (p=0.03). In contrast, patients who underwent OR exhibited a higher incidence of systemic and pulmonary complications (p<0.001 and p=0.005, respectively). Although the operative mortality was considerably higher in the OR group than in the EVAR group, with a risk ratio of 3.9, this difference did not reach statistical significance.
Greenhalgh et al. (2004) [29]	RCT	1,082	30 days	The study findings indicated that the 30-day mortality rate was notably higher in the OR group than in the EVAR group (p=0.016). Similarly, in-hospital mortality rates favored EVAR, with a significantly lower number of fatalities in the EVAR group than in the OR group (p=0.001). Furthermore, the EVAR group experienced a higher frequency of secondary interventions within 30 days, attributed to postoperative complications, including endoleaks, than the OR group (p=0.02). Notably, patients who underwent EVAR had shorter hospital stays than those who underwent OR (p<0.0001).
Greenhalgh et al. (2005) [30]	RCT	1,082	4 years	The study found no significant difference in overall mortality rates between patients who underwent OR and EVAR. However, EVAR patients had lower aneurysm-related mortality (p=0.04), shorter operative time (p<0.05), and required fewer blood products (p<0.01). In the three months post-procedure, EVAR patients reported higher QoL (p=0.01). EVAR patients had a higher rate of secondary interventions (p<0.0001), while OR patients had longer durations in the ICU and a longer overall hospital stay (p<0.05).
Patel et al. (2016) [31]	RCT	1,257	More than 8 years	The study found no statistically significant variance in overall mortality between EVAR and OR. However, aneurysm-related mortality was notably higher in the EVAR group than in the OR group (p=0.01), particularly beyond the four-year timeframe. The EVAR group exhibited a higher incidence of aneurysm rupture (p=0.0001) and a more significant requirement for secondary interventions (p=0.002) than the OR group. The average duration of the EVAR procedure was significantly shorter than that of the OR (p=0.0002). Furthermore, ICU and hospital stays were shorter for EVAR patients than for OR patients (p=0.0001 for both)
Salata et al. (2019) [32]	Comparative study	17, 683	13.8 years	The study findings indicated that EVAR resulted in higher one-year survival rates (94.0% versus 91.0%) and better significant adverse cardiovascular event-free survival rates up to four years after repair (72.9% versus 69.9%) than OR. However, the cumulative incidence of reintervention was higher among EVAR patients at the seven-year follow-up (45.9% versus 42.2%). The study did not find statistically significant differences between EVAR and OR patients in long-term survival, reintervention, or secondary rupture. The Kaplan-Meier analysis suggested improved long-term survival free of major adverse cardiovascular events in EVAR patients compared to OR patients over a maximum follow-up period of 13.8 years.
De Bruin et al. (2010) [33]	RCT	351	6 years	The study results showed no significant difference in overall survival rates between EVAR and OR. Nonetheless, in-hospital mortality rates were higher among patients undergoing OR than among those undergoing EVAR (p<0.0001). Notably, occurrences of cardiovascular-related mortalities were more prevalent in OR patients than those undergoing EVAR (p<0.05). Moreover, incidences of secondary interventions were more frequent in the EVAR cohort than in the OR cohort (p=0.03). Reinterventions post-EVAR were predominantly attributable to graft-related complications, whereas post-OR, wound-related issues, particularly incisional hernias, were more prevalent.
Lederle et al. (2019) [34]	RCT	881	14 years	The study findings highlighted the EVAR group exhibited a slightly lower overall mortality rate than the OR group, as indicated by a hazard ratio of 0.96 (p=0.61). Analysis revealed that patients undergoing OR experienced a 2.8% higher incidence of death due to pulmonary complications than those undergoing EVAR. Furthermore, within the hospital or 30 days postoperatively, OR patients had a 2.1% higher risk of aneurysm-related death than EVAR patients. In contrast, the EVAR group had a 4% higher rate of cardiovascular-related deaths than the OR group. The study also noted a higher prevalence of secondary interventions in the EVAR group than in the OR group (p=0.04). Age-stratified analysis revealed that patients over 70 had improved overall survival following OR (p=0.02), while those under 70 exhibited higher survival rates with EVAR (p=0.10).
van Schaik et al. (2017) [35]	RCT	351	15 years	The study revealed that patients who underwent EVAR had a lower mortality rate within the first six months following the operation than those who underwent OR, with an RR of 1.65 (p=0.34). However, the mortality risk was slightly lower for OR patients between six months and six years postoperatively, with an RR of 0.83. Beyond six years, the two procedures had no significant differences in mortality. Aneurysm-related deaths within the first six months postoperatively were more common in the OR group, with an RR of 3.31 (p=0.06), and this increased risk continued throughout the follow-up period, with an RR of 1.54 (p=0.35). Notably, aneurysm-related complications were more prevalent in the EVAR group, encompassing incomplete aneurysm exclusion (p<0.001) and thrombo-occlusive events (p<0.01). Conversely, incisional hernias were more frequent following OR (p=0.006). Additionally, pulmonary complications leading to mortality were significantly more frequent in the OR group (p<0.01). Finally, patients in the EVAR group required secondary interventions more frequently than those in the OR group (p<0.001).
Becquemin et al. (2011) [36]	RCT	299	3 years	The study findings indicated no significant variance in operative mortality rates between OR and EVAR. However, patients who underwent OR experienced notably prolonged hospitalization periods, necessitated a higher frequency of blood transfusions, and required ventilatory support more frequently (p<0.0001). Subsequent to EVAR, many patients necessitated secondary intervention than those who underwent OR (p<0.0001). Furthermore, postoperative buttock claudication was more prevalent in the EVAR cohort (p<0.001). Conversely, incisional and minor cardiac complication incidences were more frequently reported in the OR group (p<0.0001 and 0.05, respectively). Although the risk of postoperative sexual dysfunction was higher in the OR group, it did not attain statistical significance. The OR procedure was significantly protracted compared to EVAR (p<0.0001).
Hwang et al. (2017) [37]	RCT	240	5 years	The research findings revealed that patients undergoing non-IFU EVAR displayed the highest mortality rates at the three- and five-year follow-ups compared to OR. Patients treated with IFU EVAR demonstrated the lowest mortality rates. Secondary interventions were more prevalent in IFU EVAR and non-IFU EVAR patients than those undergoing OR (p=0.02). However, there was no statistically significant difference in secondary interventions between IFU EVAR and OR. Notably, patients in the non-IFU EVAR group showed significantly higher instances of type I endoleaks than those in the IFU EVAR group (p=0.004). Furthermore, patients undergoing non-IFU EVAR exhibited higher rates of type IA and IB endoleaks than IFU EVAR patients (p=0.162 and 0.058, respectively).
Cuypers et al. (2001) [38]	RCT	76	30 days	The study findings revealed that more patients required blood transfusions post OR than EVAR (p<0.05). Moreover, postoperative ST-segment abnormalities on ECG were greater among patients who underwent OR than those who underwent EVAR (p=0.06). Patients in the OR group exhibited an increased risk of myocardial infarction (p=0.05). Furthermore, heart wall motion anomalies on TEE were more prevalent in the OR than in the EVAR group (p<0.05). Perioperative left ventricular SWI and BP were significantly higher in the OR group, with p-values of 0.01 and 0.02. Patients who underwent EVAR experienced shorter hospital stays (p<0.01) and quicker return to regular feeding than those who underwent OR (p<0.01). Additionally, patients in the OR group had prolonged intubation periods (p<0.05) and longer ICU stays (p<0.05). They also took longer to resume regular feeding, averaging five days compared to two days for patients in the EVAR group (p<0.01).
Soulez et al. (2005) [39]	RCT	40	1 year	The study found that patients who underwent EVAR had significantly shorter stays in the ICU (p=0.0001) and hospital (p=0.001), required fewer transfusions (p=0.02), and had shorter procedure times (p=0.01). Additionally, patients in the EVAR group had fewer rehospitalizations for any cause and aneurysm-related issues (p=0.18 and 0.55, respectively) than those in the OR group. The groups had no significant differences in QoL or functional autonomy, as measured by Karnofsky and SF-36 scores. However, patients in the OR group had significantly higher morphine consumption postoperatively than those in the EVAR group (p<0.05).

Based on available clinical evidence, EVAR offers significant advantages over OR in terms of reduced invasiveness, lower operative stress, shorter hospital stays, and quicker recovery times. However, the long-term outcomes of EVAR remain a concern due to the higher rates of secondary interventions and potential complications such as endoleaks. Despite these concerns, EVAR has emerged as the preferred surgical option for AAA treatment, particularly in patients with suitable anatomy. The decision between EVAR and OR should be individualized, considering patient-specific factors, anatomical considerations, and the availability of experienced surgical teams. In practice, the choice of procedure may depend on various factors, including patient preference, risk assessment, and the surgical team's expertise.

Discussion

The comparative efficacy and safety of EVAR and OR for AAA treatment have improved significantly over the past two decades. In this discussion, we delve into the perioperative and short-term outcomes, long-term efficacy and mortality, secondary interventions, cardiac and pulmonary complications, and the impact on QoL and functional autonomy. By analyzing the findings of various studies in this review, we aim to provide a comprehensive overview of the advantages and potential trade-offs associated with EVAR and OR, shedding light on the key considerations vital for clinical decision-making and patient care.

Perioperative and Short-Term Outcomes

Prinssen et al. provided an early indication of EVAR's advantages in perioperative settings, demonstrating significantly shorter operation times, reduced intraoperative blood loss, fewer blood transfusions, and lower rates of mechanical ventilation than those associated with OR. Although the difference in operative mortality between the two groups was not statistically significant, EVAR patients had a lower mortality rate, suggesting a potential survival benefit in the short term [28]. Reinforcing these findings, Greenhalgh et al. reported a markedly lower 30-day mortality rate for EVAR in a larger cohort. Additionally, EVAR patients experienced shorter hospital stays and procedure durations, further solidifying EVAR's role in improving immediate postoperative recovery. However, the increased frequency of secondary interventions in the EVAR group highlights a critical trade-off, necessitating a balance between short-term benefits and the potential for subsequent complications [29].

Long-Term Efficacy and Mortality

The long-term follow-up study by Greenhalgh et al. provided valuable insights into the durability of EVAR outcomes. While there were initial advantages concerning QoL, reduced blood product use, and shorter hospital stays, the study revealed no significant difference in overall mortality between EVAR and OR after four years. However, EVAR was associated with lower aneurysm-related mortality, indicating a sustained benefit in preventing aneurysm rupture. The study also noted higher long-term costs related to EVAR due to the need for ongoing surveillance and secondary procedures [30]. This was further corroborated by Patel et al., who observed a slightly higher but non-significant increase in overall mortality with EVAR beyond four years, coupled with a significantly higher aneurysm-related mortality. These findings suggest that while EVAR offers compelling short-term advantages, its long-term efficacy remains contingent upon vigilant follow-up and management of complications [31].

Secondary Interventions and Complications

The propensity for EVAR to necessitate more frequent secondary interventions is a recurring observation in the literature. Salata et al. and De Bruin et al. highlighted the higher incidence of secondary interventions required in EVAR patients, particularly related to graft complications [32-33]. Despite these challenges, Salata et al. noted higher survival rates up to one year post-repair and superior event-free survival for up to four years with EVAR [32]. These findings were consistent with De Bruin et al., which showed no significant difference in overall survival between EVAR and OR over six years but noted lower in-hospital mortality rates and cardiovascular-related deaths in the EVAR group [33]. These observations underscore the importance of manufacturer specifications and careful patient selection in optimizing EVAR outcomes, as emphasized by Hwang et al., who found that EVAR procedures performed in compliance with manufacturer guidelines had the lowest mortality rates relative to non-compliant EVAR procedures [37].

Cardiac and Pulmonary Complications

Cuypers et al. provided an essential perspective on the cardiac complications associated with EVAR and OR. Their findings indicated that OR patients had a higher incidence of postoperative ECG abnormalities, myocardial infarction, and extended ICU stays, which were attributed to the more invasive nature of the procedure. In contrast, EVAR patients experienced shorter hospital stays, quicker recovery times, and required fewer blood transfusions [38]. These results aligned with Becquemin et al., who reported more extended hospital stays, increased ventilatory support, and higher transfusion requirements in OR patients. EVAR has an overall safety profile that minimizes cardiac and pulmonary complications, making it a viable, less-invasive alternative to OR [36].

QoL and Functional Autonomy

The assessment of QoL and functional autonomy provides a broader context for evaluating the impact of EVAR and OR on patient well-being. Soulez et al. conducted a randomized controlled trial comparing these outcomes and found no significant differences in QoL or functional autonomy between the two procedures. However, EVAR patients benefited from quicker procedure times, shorter ICU and hospital stays, and required fewer transfusion needs. The higher postoperative morphine consumption in OR patients suggests more significant pain and reflects potentially prolonged recovery periods. These findings contrast with the reduced invasiveness of EVAR, which appears to offer comparable long-term QoL and functional autonomy to OR while minimizing postoperative discomfort [39].

## Conclusions

The comparative analysis of EVAR and OR for treating AAA patients reveals compelling insights into their efficacy and safety profiles. Perioperative and short-term outcomes demonstrate the advantages of EVAR in terms of reduced operation times, blood loss, blood transfusions, and mechanical ventilation requirements compared to OR. Additionally, EVAR patients experience shorter hospital stays and procedure durations, contributing to improved immediate postoperative recovery. Long-term efficacy and mortality considerations show no significant difference in overall mortality between EVAR and OR. However, EVAR demonstrates potential sustained benefits in preventing aneurysm rupture, albeit with higher long-term costs due to ongoing surveillance and secondary procedures. While EVAR may require more frequent secondary interventions relative to OR, it has higher survival rates and superior event-free survival up to four years post-repair. Cardiac and pulmonary complications analysis indicates that EVAR has an overall safety profile, minimizing associated complications and making it a viable, less-invasive alternative to OR. It is evident that while EVAR offers compelling short-term advantages, its long-term efficacy remains contingent upon vigilant follow-up and management of complications. Therefore, when considering the choice between EVAR and OR for AAA treatment, weighing the short-term benefits of EVAR against potential long-term considerations such as surveillance, costs, and the need for secondary interventions is imperative. This comprehensive understanding of the advantages and potential trade-offs associated with EVAR and OR is vital for informed clinical decision-making and patient care.

## Appendices

("Aortic Aneurysm, Abdominal"[Mesh]) AND ( "Aortic Aneurysm, Abdominal/classification"[Mesh] OR  "Aortic Aneurysm, Abdominal/diagnosis"[Mesh] OR  "Aortic Aneurysm, Abdominal/diagnostic imaging"[Mesh] OR  "Aortic Aneurysm, Abdominal/pathology"[Mesh] OR  "Aortic Aneurysm, Abdominal/surgery"[Mesh] )

("Aortic Aneurysm, Abdominal"[Mesh]) AND "Aortic Aneurysm, Abdominal/surgery"[Mesh]

(("Aortic Aneurysm, Abdominal"[Mesh]) AND "Vascular Surgical Procedures"[Mesh]) AND "Endovascular Aneurysm Repair"[Mesh]

((("Aortic Aneurysm, Abdominal"[Mesh]) AND "Aortic Aneurysm, Abdominal/surgery"[Mesh]) AND "Endovascular Aneurysm Repair"[Mesh]) AND "Endovascular Procedures"[Mesh]
